# An age-related shift of resting-state functional connectivity of the subthalamic nucleus: a potential mechanism for compensating motor performance decline in older adults

**DOI:** 10.3389/fnagi.2014.00178

**Published:** 2014-07-23

**Authors:** Christian Mathys, Felix Hoffstaedter, Julian Caspers, Svenja Caspers, Martin Südmeyer, Christian Grefkes, Simon B. Eickhoff, Robert Langner

**Affiliations:** ^1^Department of Diagnostic and Interventional Radiology, Medical Faculty, Heinrich Heine University DüsseldorfDüsseldorf, Germany; ^2^Institute of Clinical Neuroscience and Medical Psychology, Medical Faculty, Heinrich Heine University DüsseldorfDüsseldorf, Germany; ^3^Institute of Neuroscience and Medicine (INM-1, INM-3), Research Centre JülichJülich, Germany; ^4^Center for Movement Disorders and Neuromodulation, Department of Neurology, Medical Faculty, Heinrich Heine University DüsseldorfDüsseldorf, Germany; ^5^Neuromodulation and Neurorehabilitation Group, Max Planck Institute for Neurological ResearchCologne, Germany; ^6^Department of Neurology, University of CologneCologne, Germany

**Keywords:** subthalamic nucleus, resting-state fMRI, functional connectivity, healthy aging, age-related changes

## Abstract

Healthy aging is associated with decline in basic motor functioning and higher motor control. Here, we investigated age-related differences in the brain-wide functional connectivity (FC) pattern of the subthalamic nucleus (STN), which plays an important role in motor response control. As earlier studies revealed functional coupling between STN and basal ganglia, which both are known to influence the conservativeness of motor responses on a superordinate level, we tested the hypothesis that STN FC with the striatum becomes dysbalanced with age. To this end, we performed a seed-based resting-state analysis of fMRI data from 361 healthy adults (mean age: 41.8, age range: 18–85) using bilateral STN as the seed region of interest. Age was included as a covariate to identify regions showing age-related changes of FC with the STN seed. The analysis revealed positive FC of the STN with several previously described subcortical and cortical regions like the anterior cingulate and sensorimotor cortex, as well as not-yet reported regions including central and posterior insula. With increasing age, we observed reduced positive FC with caudate nucleus, thalamus, and insula as well as increased positive FC with sensorimotor cortex and putamen. Furthermore, an age-related reduction of negative FC was found with precuneus and posterior cingulate cortex. We suggest that this reduced de-coupling of brain areas involved in self-relevant but motor-unrelated cognitive processing (i.e. precuneus and posterior cingulate cortex) from the STN motor network may represent a potential mechanism behind the age-dependent decline in motor performance. At the same time, older adults appear to compensate for this decline by releasing superordinate motor control areas, in particular caudate nucleus and insula, from STN interference while increasing STN-mediated response control over lower level motor areas like sensorimotor cortex and putamen.

## INTRODUCTION

The proportion of the world’s population over 60 years of age will presumably double from about 11–22% (i.e., amounting to 2 billion people) between the years 2000 and 2050^1^. There is thus a need for a more profound comprehension of the psychological and physiological changes associated with healthy aging.

A typical feature of aging is a deterioration of motor control (Seidler et al., 2010), the neural substrates of which have recently moved into the focus of research (Heuninckx et al., 2005; Ward et al., 2008; Harada et al., 2009; Seidler et al., 2010; Noble et al., 2011; Goble et al., 2012). For instance, it was shown that deterioration of motor performance in advanced age is associated with a more widespread involvement of prefrontal cortex and basal ganglia, which might reflect an increased reliance on cognitive control mechanisms, in compensation for lower level sensorimotor processing deficits (Seidler et al., 2010).

A key structure that is connected to both motor system and cognitive areas is the subthalamic nucleus (STN, Hamani et al., 2004; Temel et al., 2005). The STN is an integral part of current models of basal ganglia motor functions, which differentiate direct and indirect striatal output pathways, originating from different neuronal populations and which are characterized by opposite end-effects (Weintraub and Zaghloul, 2013). The direct pathway represents inhibitory circuits from the striatum to the internal segment of the globus pallidus, leading to a decreased inhibition of the ventrolateral thalamic nucleus, which in turn is associated with motor facilitation (Weintraub and Zaghloul, 2013). In contrast, the STN is a key structure of the polysynaptic indirect pathway. In addition to inhibitory striatal afferents, the internal segment of the globus pallidus is stimulated by the STN, which then leads to motor inhibition via an increased inhibition of the ventrolateral thalamus (Alexander and Crutcher, 1990). Both STN and striatum receive excitatory input from primary motor cortex (M1), premotor cortex (PMC), and primary somatosensory cortical (S1) areas (Weintraub and Zaghloul, 2013). Previous studies on STN functional connectivity (FC) based on resting-state (RS) functional magnetic resonance imaging (fMRI; Baudrexel et al., 2011; Brunenberg et al., 2012) as well as on its anatomical connectivity using diffusion-weighted imaging (DWI; Lambert et al., 2012) showed bilateral coupling with the striatum including caudate nucleus, putamen, and globus pallidus, as well as midbrain, thalamus, dorsal pontine areas, cerebellum, supplementary motor area (SMA) cortex, and lateral PMC. The STN’s involvement in motor functions is further corroborated by marked improvements in motor functioning after electrical stimulation of the STN in Parkinson’s disease (PD; Limousin et al., 1995).

Key functions of the STN in the context of motor control include the control of response thresholds in the fronto-striatal network (Mansfield et al., 2011), thereby playing a role in stopping of ongoing motor activity (Aron et al., 2007) and inhibiting responses during conflict (Frank et al., 2007; Brittain et al., 2012). According to recent models, competing cortical input from a “hyperdirect pathway” (excitatory projection from PMC and M1 to STN) is integrated in the STN to dynamically adjust the response threshold (Nambu et al., 2002; Frank, 2006). It is important to appreciate, however, that the STN has not only been implicated in motor control but has also been related to affective and cognitive processes, given that it is connected to key regions of these systems (Hamani et al., 2004; Temel et al., 2005). Thus, the STN seems to be one of the gateways between motor control and cognition.

Given age-related changes in cognitive and motor performance as well as their integration (for a review, see Seidler et al., 2010), the location of the STN at the interface of these networks raises the question of how this STN network may change in the course of healthy aging. Besides changes in regional brain structure and activity, differences in interregional coupling patterns have been increasingly recognized as a neural substrate of behavioral changes with age (Lee et al., 2006; Madden et al., 2010; Langner et al., 2014). Such age-related FC changes have shown to occur independently from structural changes (Hoffstaedter et al., 2014; Langner et al., 2014) and to predict behavioral changes, even in the absence of changes in regional activation (Grady, 2005; Madden et al., 2010). Therefore, in this study, we aimed to examine age-related changes of STN FC. Given previous evidence for increased reliance on cognitive control mechanisms in the context of age-related deficits in lower level motor control (Seidler et al., 2010), we expect a shift in functional integration of the STN with the striatum in the elderly, since both regions are known to influence the cognitively mediated conservativeness of motor responses (Mansfield et al., 2011). We further hypothesized that RS FC of the STN with the striatum becomes dysbalanced with age.

## MATERIALS AND METHODS

### SEED DEFINITION

**FIGURE 1:**
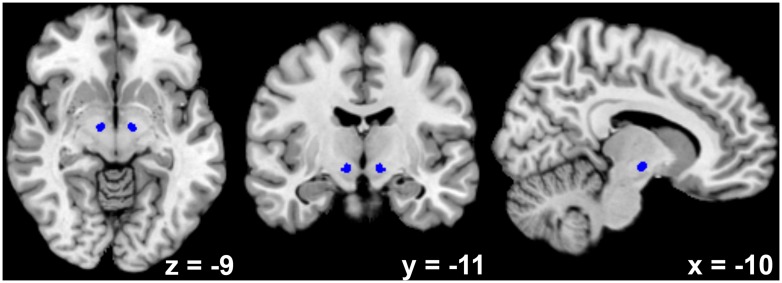
**Depiction of the bilateral subthalamic nucleus seed region as derived from high-resolution susceptibility mapping using 7-T magnetic resonance imaging (Schafer et al., 2012)**.

Bilateral STN seed regions of interest (ROIs; see **Figure 1**) were derived from a recently published atlas that was created using high-resolution susceptibility mapping of this brain area in eight healthy individuals using 7-T MRI (Schafer et al., 2012). Previous studies have reported a slight shift of the STN in lateral direction with increasing age (Keuken et al., 2013). However, we consider this shift irrelevant for the present study because the extent of the Gaussian kernel we used for spatial smoothing (5 mm) clearly exceeded the size of the observed shift (≈2 mm). For the main network analysis, the activation time series (first eigenvariate) of both left and right STN ROIs were collapsed into a common seed to increase the reliability of the analysis. In a supplementary analysis of hemispheric differences, the two ROIs were used as separate seeds.

### SAMPLE

The sample included RS fMRI data from 361 healthy adults(34.5%♀) with a mean age of 41.9 (SD = 16.5, median = 41, interquartile range = 28) years. All volunteers gave written informed consent to the study and were without any record of neurological or psychiatric disorder. Three different sites contributed data (see **Table 1**). Approval for the joint (re-)analysis was obtained from the local ethics committee of the University Hospital Düsseldorf.

**Table 1:** Characteristics of the sample.

Contribution Site	*n*	Mean age (range)	Sex: male (%)	Measurement parameters^a^
RWTH University Hospital Aachen, Germany	47	36.5 (19–59)	46	3 T/250/2.2/30/80°/3.1 mm× 3.1 mm× 3.1 mm
	28	63.4 (55—72)	71	3 T/270/2.2/30/90°/3.1 mm× 3.1 mm× 3.1 mm
Research Centre Jülich, Germany	54	28.5 (18–59)	54	3 T/250/2.2/30/90°/3.1 mm× 3.1 mm× 3.1 mm
	100	45.1 (21–71)	49	3 T/300/2.2/30/90°/3.1 mm× 3.1 mm× 3.1 mm
NKI/Rockland, Orangeburg, NY, USA^b,c^	132	42.3 (18–85)	59	3 T/260/2.5/30/80°/3.0 mm× 3.0 mm× 3.0 mm

*NKI, Nathan S. Kline Institute.*

a Measurement parameters: magnetic field strength of the scanner/number of acquired volumes/repetition time (in s)/echo time (in ms)/flip angle/voxel size.

b These data were selected from the datasets included in Biswal et al. (2010) and made publicly available via the 1000 Functional Connectomes Project http://fcon_1000.projects.nitrc.org.

c All but the participants of the NKI/Rockland sample were instructed to keep their eyes closed during the measurement.

### DATA ACQUISITION AND PREPROCESSING

Echo-planar imaging (EPI) of the entire brain (vertex to lower parts of the cerebellum) at 3 T was used to record >9 min of blood oxygen level-dependent (BOLD) activity in all participants (detailed measurement parameters are provided in **Table 1**). The scan parameters of the three different sites were not identical but similar (see **Table 1**). Site effects were also removed in the group-level data analysis (see below). Participants were instructed to let their mind wander and not to fall asleep. Joint preprocessing of all data was performed with SPM8^2^. The first four images of each BOLD time series were discarded to account for saturation effects. A two-pass affine registration procedure was used for motion correction. The images were initially realigned to the first image and afterwards to the mean of the realigned images. The mean image was also used for normalization to the Montreal Neurological Institute (MNI) single-subject template as provided in SPM8, using the unified segmentation approach (Ashburner and Friston, 2005). The same normalization parameters were then applied to each individual EPI volume. During normalization, image volumes were resampled to a voxel size of 1.5 mm× 1.5 mm× 1.5 mm. A 5-mm full-width at half-maximum Gaussian kernel was subsequently applied to spatially smooth the images.

### DATA ANALYSIS

We used a validated protocol (Satterthwaite et al., 2013b) that has been proven to reliably detect age-related FC-changes, which is especially important in the context of known correlations between age and in-scanner motion. Accordingly, to avoid correlations explained by nuisance variables, variance explained by the following parameters was removed (Jakobs et al., 2012; Satterthwaite et al., 2013a): (i) the six motion parameters obtained from spatial realignment, (ii) the first derivates of the six motion parameters, (iii) the time series of the gray-matter, white-matter, and cerebrospinal-fluid signal intensity obtained by averaging, at each point in time, across all voxels of the respective tissue class (as determined by the SPM8 segmentation). In order to remove residual low- and high-frequency noise, the data were band-pass filtered preserving frequencies between 0.01 and 0.08 Hz (Biswal et al., 1995; Greicius et al., 2003; Fox and Raichle, 2007).

The time series of both STNs, represented by the first eigenvariate of all STN voxels’ time series, was then correlated with the time series of all other gray-matter voxels, and the resulting Pearson correlation coefficients were transformed into Fisher’s *Z* scores for every voxel. In the subsequent group-level analysis of variance (ANOVA), sex, data contribution site (three subsamples), and age were used as covariates.

Age effects on STN FC were tested in conjunction with the respective FC main effect. For example, age-related decreases of positive FC were tested using the following conjunction: [positive FC with bilateral STN] ∩ [age-related decrease of FC with bilateral STN]. For the supplementary analysis of hemispheric differences between left and right STN, the same approach was adopted. Results were considered significant at cluster-level *p* < 0.05 (family-wise error-corrected for multiple comparisons; cluster-forming threshold at voxel level: *p* < 0.001). For exploratory purposes, a more liberal cluster-level threshold of *p* < 0.1 was also adopted for the analysis of age-dependent decline in positive FC of STN. Resulting brain areas were anatomically assigned via the SPM Anatomy toolbox^3^ (Eickhoff et al., 2007), which implemented probabilistic cytoarchitectonic mapping (Zilles and Amunts, 2010).

A split-half group comparison between the 180 younger und 180 older participants was also performed. Clusters for age effects and hemispheric differences are only reported if their time series show a correlation of at least *r* > 0.1 or *r* < -0.1 (equivalent to at least a small effect size according to Cohen’s definition, Cohen, 1988) with the STN time series for either the 180 younger or the 180 older participants or both.

## RESULTS

### WHOLE-BRAIN FC PATTERN OF THE STN

**FIGURE 2:**
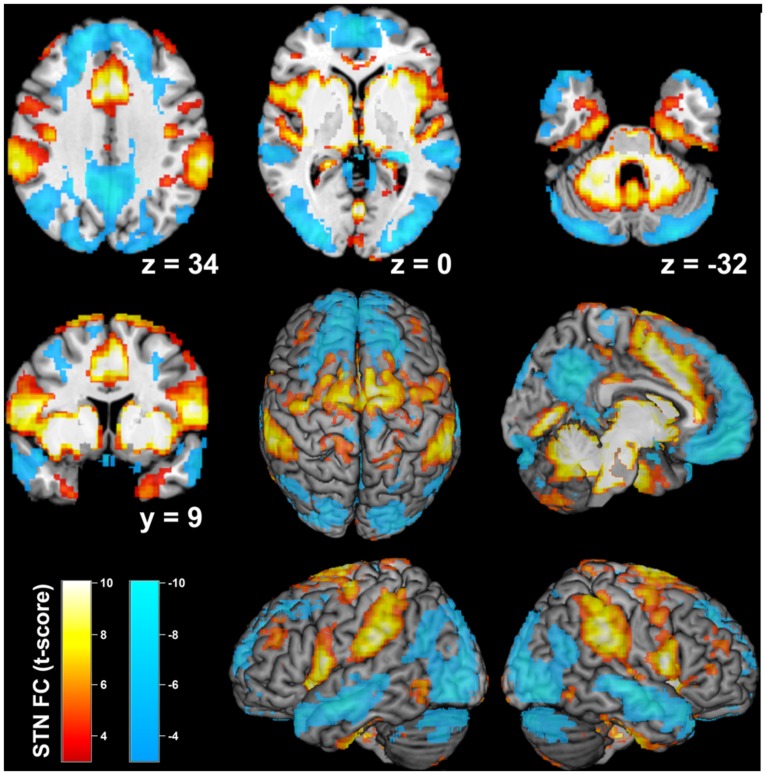
**Resting-state functional connectivity of the bilateral subthalamic nucleus seed region.** Positive network coupling is denoted in warm colors, while cold colors denote anticorrelated regions. Montreal Neurological Institute coordinates are provided for transsectional slices. Surface rendering was performed with MRIcron (Rorden and Brett, 2000).

Bilateral STN RS-FC analysis revealed a significantly positive coupling between STN and the following areas bilaterally (**Figure 2**): putamen, caudate nucleus, external and internal globus pallidus (GPe and GPi), thalamus (prefrontal, temporal, parietal, premotor, somatosensory and motor. Behrens et al., 2003), anterior and midcingulate cortex, posterior (Kurth et al., 2010a) and central insula extending to the parietal operculum (areas OP 1–4, Eickhoff et al., 2006a,b), inferior frontal gyrus (areas 44/45, Amunts et al., 1999, 2004), dorsolateral prefrontal cortex, PMC (area 6, Geyer, 2004), S1 and M1 (areas 1, 2, 3a, 3b, and 4, Geyer et al., 1996, 1999, 2000; Grefkes et al., 2001; Geyer, 2004), rostral superior parietal cortex (area 5Ci, Scheperjans et al., 2008a,b), rostral inferior parietal cortex (PF, PFm, PFt, PFop, PFcm, Caspers et al., 2006, 2008), primary visual cortex (V1; area 17, Amunts et al., 2000), hippocampus (area EC, Amunts et al., 2005), cerebellum (lobules I–VI, Diedrichsen et al., 2009), midbrain, and pons.

**FIGURE 3:**
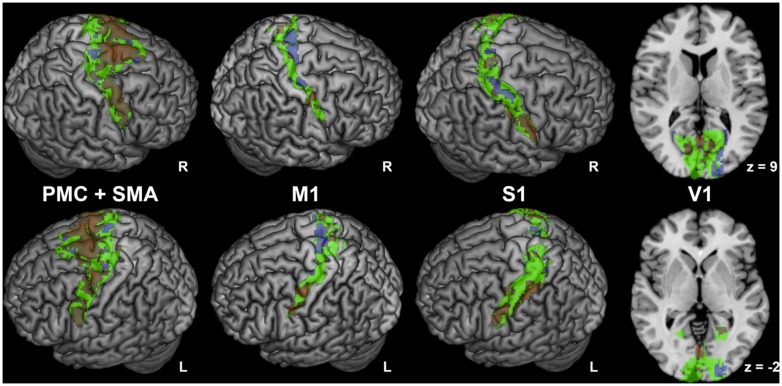
**Regions with mixed connectivity pattern to subthalamic nucleus.** Different fractions of premotor corex and supplementary motor area (PMC and SMA; Geyer, 2004), primary motor cortex (M1; Geyer et al., 1996), primary sensory cortex (S1; Geyer et al., 1999, 2000; Grefkes et al., 2001) and primary visual cortex (V1; Amunts et al., 2000) showing positive or negative functional connectivity with the subthalamic nucleus. Cytoarchitectonic regions are denoted in green, positive network coupling in warm colors, and anticorrelated network coupling in cold colors. Correlated and anticorrelated clusters do not overlap; this impression is an artefactual result of the surface rendering of 3-D clusters.

Anticorrelated regions (**Figure 2**) comprised bilateral orbitofrontal cortex, PMC and M1 (area 4, Geyer et al., 1996), posterior cingulate cortex, precuneus, posterior S1 (area 2, Grefkes et al., 2001), caudal superior parietal cortex (area 7a, Scheperjans et al., 2008a,b), caudal inferior parietal lobule (PGa, Caspers et al., 2006, 2008), V1 and secondary visual cortex (Amunts et al., 2000), cerebellum (lobules VIIa, IX, Diedrichsen et al., 2009). Anticorrelated regions were also observed in bilateral hippocampus (area CA, SUB, and FD Amunts et al., 2005) and amygdala (area LB and SF, Amunts et al., 2005). Interestingly, different fractions of M1, PMC, S1, and V1 showed positive or negative FC with STN, respectively (**Figure 3**).

In the PMC and SMA, we found a considerably larger proportion of voxels with positive than negative FC with the seed (**Figure 3**). While positively correlated voxels were distributed across the whole PMC and SMA regions, negatively correlated voxels were mostly located medially at the anterior and posterior border of the SMA. In M1, we found moderately more negatively correlated voxels (**Figure 3**), which were predominantly found medially in the anterior part of Area 4 (Area 4a, Geyer et al., 1996), while positively correlated clusters were found laterally and medially in its posterior part (Area 4p, Geyer et al., 1996).

In the posterior S1, we found moderately more voxels positively correlated with STN; they were more numerous in the lateral part, while anticorrelated voxels were mostly located medially and were more pronounced on the right side (see **Figure 3**).

In V1, voxels showing positive FC with STN were located bilaterally in the rostral part, while anticorrelated voxels were nearly exclusively found in the right-sided occipital part of this region.

### AGE EFFECTS ON THE FC PATTERN OF THE STN

**FIGURE 4:**
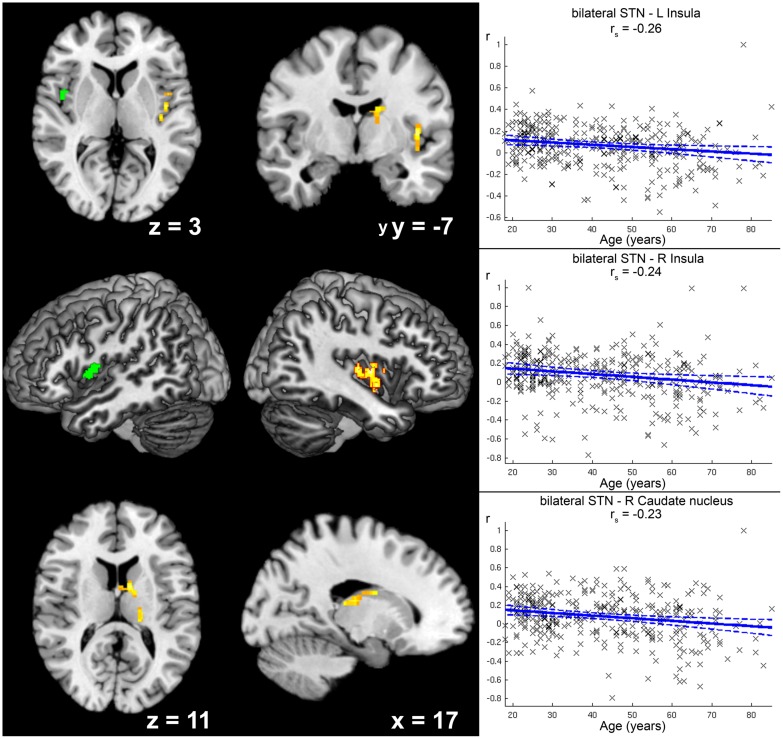
**Age-related decrease in positive functional connectivity (FC) with the subthalamic nucleus (STN).** Clusters showing a significant age-related decrease in positive functional FC with the seed region (i.e., bilateral STN) are marked in warm colors, while the green cluster was only marginally significant (*p* = 0.072) after correction for multiple testing. Coordinates refer to Montreal Neurological Institute space. Surface reconstructions of both hemispheres were cropped to allow visibility of insular clusters. Scatter plots for each of the three clusters show the individual FC values against age across all participants. Linear regression lines with 95% confidence interval (dotted lines), as well as *r*_s_-values (Spearman’s rank correlation coefficient) are provided for each significant cluster, respectively.

There were several differences in RS-FC related to age (see **Table 2**): Age-dependent decline (**Figure 4**) in positive FC of STN was found within the caudate nucleus of the right striatum, right thalamus (prefrontal and parietal zone, Behrens et al., 2003), and right central and posterior insula (Ig2, Kurth et al., 2010a). There was a trend toward significance for a cluster within the left central insula (*p* = 0.072).

**Table 2:** Adult age differences in intrinsic functional connectivity between both STNs and associated brain regions.

Pair of regions	Coordinates*x*/*y*/*z* (mm)	Mean *r*_young_	Mean *r*_elderly_	*T*-scoreΔ*r*	Corr. with age (*r*_s_)
L STN–R STN	N/A	0.388	0.474	-1.82	0.17
STN–L posterior insula	-45/2/4	0.133	0.014	2.85**	-0.26
STN–R posterior insula	38/-18/6	0.152	0.001	2.58*	-0.24
STN–R caudate nucleus/thalamus	16/-6/20	0.138	-0.004	3.08**	-0.23
STN–R sensorimotor	42/-14/34	0.028	0.141	-4.44***	0.28
STN–L putamen	-16/11/-6	0.094	0.232	-3.13**	0.21
STN–L precuneus/PCC	-8/-56/42	-0.107	0.011	-5.33***	0.28
STN–R V3/V4	34/-83/-12	-0.037	-0.119	1.90	-0.23

Mean *r*_young_ and *r*_elderly_ denote group-averaged functional connectivity (FC) in the 180 youngest and 180 oldest participants, respectively; Δ*r T*-score denotes the *T*-statistic (*p < 0.05; **p < 0.01; ***p < 0.001) for the comparison of the two FC-values between both subgroups.

**FIGURE 5:**
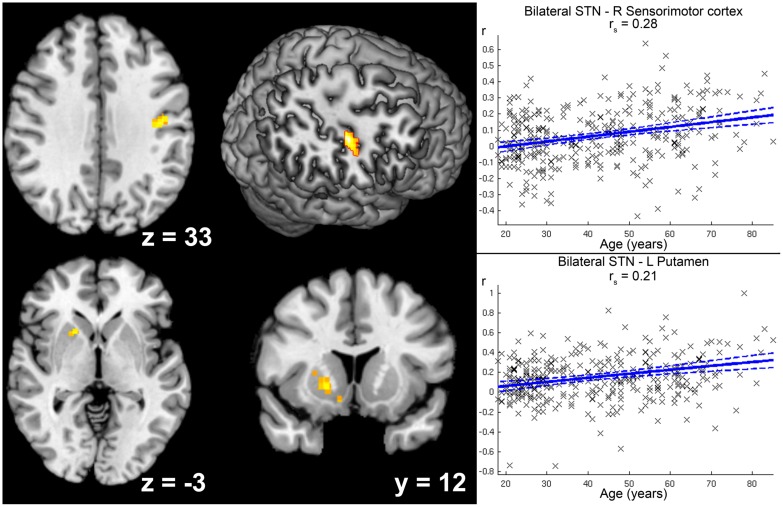
**Age-related increase in positive functional connectivity (FC) with the subthalamic nucleus (STN).** Clusters showing a significant age-related increase in positive FC with the seed region (i.e., bilateral STN) are marked in warm colors. Coordinates refer to Montreal Neurological Institute space. The second image in the first row shows a right-sided ascending (45°) view on a surface reconstruction of the brain. Parts of the right central region have been cropped away to allow visibility of the cluster in right sensorimotor cortex. Scatter plots for each of the two clusters show the individual FC values against age across all participants. Linear regression lines with 95% confidence interval (dotted lines), as well as *r*_s_-values (Spearman’s rank correlation coefficient) are provided for each significant cluster, respectively.

Increases in positive STN FC with age were found for right sensorimotor cortex (areas 3a, 4p) and left putamen (see **Figure 5**).

**FIGURE 6:**
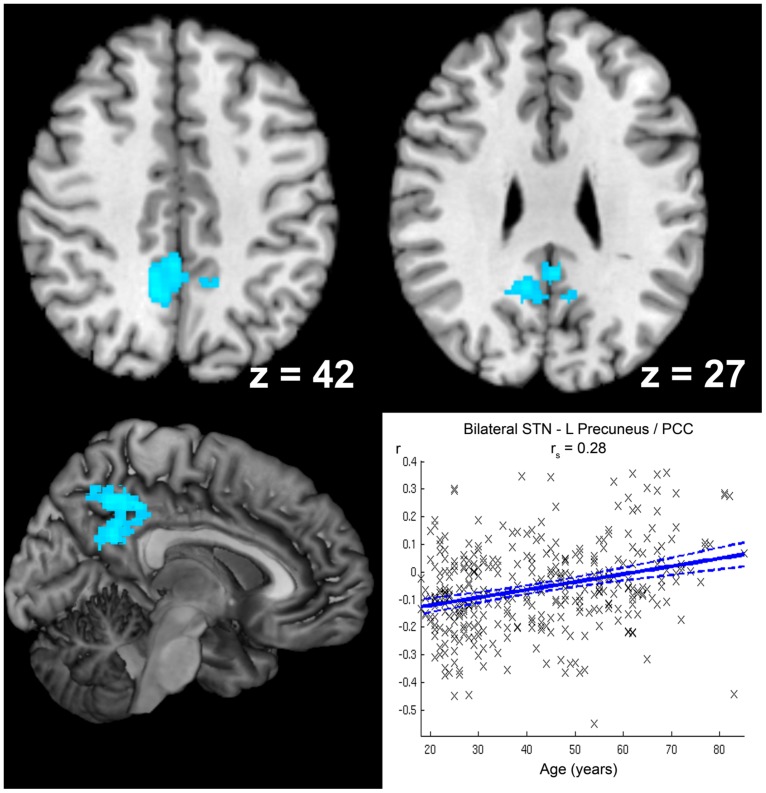
**Age-related decrease in anticorrelation with the subthalamic nucleus (STN).** The cluster showing a significant age-related decrease in anticorrelation with the seed region (i.e., bilateral STN) is marked in cold colors. Coordinates refer to Montreal Neurological Institute space. A scatter plot shows the individual correlation values of this cluster against age across all participants. Linear regression lines with 95% confidence interval (dotted lines), as well as *r*_s_-values (Spearman’s rank correlation coefficient) are provided.

Age-related decrease in negative STN FC (i.e., diminished anti-correlation with age; see **Figure 6**) was detected for left precuneus extending to posterior cingulate cortex, while stronger anti-correlation with age (see **Table 2**) was observed for right ventral extrastriate cortex (area V3/V4, Rottschy et al., 2007).

### SUPPLEMENTARY ANALYSIS OF HEMISPHERIC DIFFERENCES IN AGE-RELATED FC CHANGES

**FIGURE 7:**
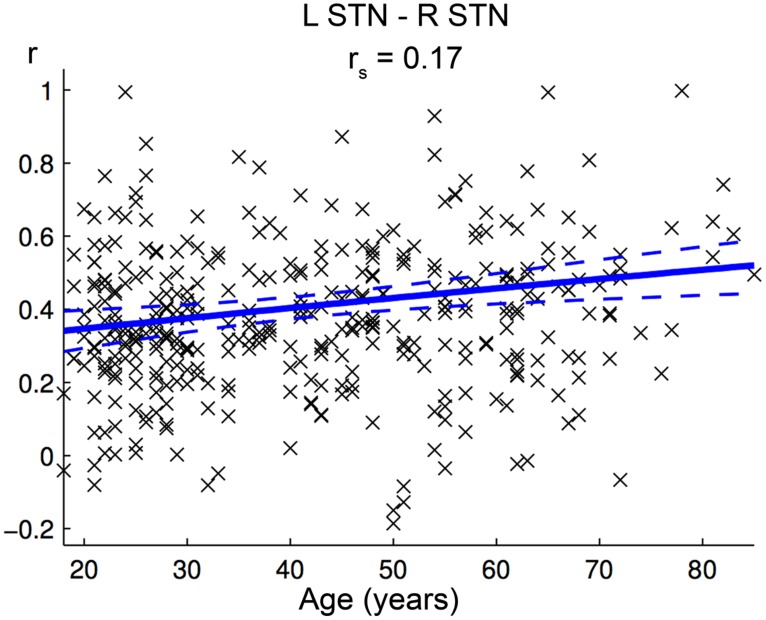
**Age-effect on interhemispheric functional connectivity (FC) between both subthalamic nuclei (STN).** The scatter plot shows the individual FC values between both STN against age across all participants. Linear regression lines with 95% confidence interval (dotted lines), as well as *r*_s_-values (Spearman’s rank correlation coefficient) are provided.

Positive FC between left and right STN increased significantly with age (**Figure 7**, **Table 2**). There were no clusters in the brain that showed a significantly stronger positive or negative FC with either the left or right STN. Also, no hemispheric differences were found for the eight possible combinations of age-related increase/decline, positive/negative FC, and preponderance for left/right STN.

## DISCUSSION

In this RS fMRI study in 361 participants an atlas-based analysis (SPM Anatomy toolbox V1.8 with implemented probabilistic cytoarchitectonic mapping, Eickhoff et al., 2007; Zilles and Amunts, 2010) revealed complex connectivity patterns, with positive and negative FC to the STN for the PMC, M1, S1, and V1. Moreover, we found several age-related changes of FC with STN. According to our hypothesis, the STN’s FC with the caudate nucleus and also with the central insula declined with age, but at the same time increased with the putamen and also right M1. Notably, anti-correlations with STN decreased with age for the precuneus and left posterior cingulate cortex, while they increased with age for right ventral extrastriate cortex.

### FUNCTIONAL CONNECTIVITY OF THE STN

The pattern of RS-FC of bilateral STN in our sample is largely in line with previous reports on STN connectivity, comprising RS-FC analysis, DWI-based studies, tract tracing studies in animals and positron emission tomography (PET) studies (Aravamuthan et al., 2007; Le Jeune et al., 2010; Baudrexel et al., 2011; Brunenberg et al., 2012; Lambert et al., 2012; Manes et al., 2013). After deep brain stimulation (DBS) of the STN in Parkinson patients, PET studies revealed decreased glucose metabolism in brain areas that were reported to show positive coupling with STN (Le Jeune et al., 2010). This was in line with the observation that high-frequency stimulation of the STN reduces firing rate and oscillatory activity of STN neurons and in the STN network (Meissner et al., 2005).

The presence of FC between the STN and basal ganglia as well as thalamus is in strong concordance with current models of basal ganglia anatomy and physiology (see Introduction). Also, the activation of anterior and midcingulate cortex has repeatedly been associated with attention and related motor control functions, such as output-related attention, preparation, and response selection (Devinsky et al., 1995; Jueptner et al., 1997; Rubia et al., 1999; Langner et al., 2011, 2012; Cieslik et al., 2013; Hoffstaedter et al., 2013a,b). In concordance with these functions, connectivity between cingulate cortex and basal ganglia has been previously described (Margulies et al., 2007; Beckmann et al., 2009; Kelly et al., 2009; Hoffstaedter et al., 2013a). The inclusion of the STN in this network appears reasonable, because the aforementioned cognitive functions involve the inhibition of certain responses in favor of others, which is one of the presumed functions of the STN (Frank et al., 2007; Brittain et al., 2012).

Besides positive FC, we found functional anticorrelations (i.e., neural de-coupling) between STN and posterior cingulate cortex and precuneus. This agrees well with previous reports on the FC pattern of the STN (Baudrexel et al., 2011). DWI studies also suggested a significant structural connectivity of STN and posterior cingulate gyrus (Brunenberg et al., 2012; Lambert et al., 2012). Along the same lines, PET scans after STN inhibition by DBS showed an increased glucose metabolism for posterior cingulate gyrus on both sides (Le Jeune et al., 2010), which is consistent with the observed functional anticorrelation. Since posterior cingulate cortex and precuneus are essentially related to non-motor functions (e.g. introspection, as discussed in more detail below), de-coupling of these areas from the STN’s motor network appears very reasonable. As for any non-motor cognitive functions of the STN, it seems that the networks involved in these functions are most likely not coupled to the posterior cingulate cortex and precuneus, which are also known to be part of the default-mode network (Leech and Sharp, 2013).

Some regions showed a mixed connectivity pattern, with different subregions showing positive or negative FC with STN. PMC and SMA were mostly positively correlated with the STN. Although clear distinction from neighboring regions (especially M1) is difficult, some anticorrelated clusters were found in the SMA. It is unclear whether the STN drives PMC and SMA or, vice versa, the STN is driven by these regions. However, in case of a direct influence from STN on PMC and SMA, pathological hyperactivity of the STN in PD patients could interfere with most of both areas, but parts of the SMA region appear to be excluded from this interference and may thus have higher chances to be part of compensation mechanisms. Correspondingly, recent reports of increased SMA-M1 coherence in magnetoencephalography data of PD patients have been interpreted as a compensatory mechanism (Pollok et al., 2013).

In M1, positively correlated voxels accumulated laterally at the face representation (Meier et al., 2008). Correspondingly, reduction of glucose metabolism in PET scans after STN inhibition by DBS was more pronounced in the lateral aspects of M1 (Le Jeune et al., 2010). To the best of our knowledge, there is no further evidence in the literature for the possible conclusion that the STN’s control over facial motor functions could be stronger than over extremity functions. On the other hand, the STN’s particular functional connection to facial motoneurons might represent a substrate for apraxia of lid opening, which is a typical side effect after DBS in PD patients (Tommasi et al., 2012) and might indicate co-stimulation and consecutive dysfunction of STN-connected regions.

Within the STN’s elaborate functional network of correlated and anticorrelated brain areas, we found several age-related changes, which will be discussed in the following section.

### AGE-RELATED CHANGES OF POSITIVE FC OF THE STN

According to our hypothesis, the STN’s positive FC with the striatum declined with age in the caudate nucleus and also in the insula, but at the same time, increased with age in the putamen and also in M1.

According to a modular conception of the striatum, the caudate nucleus is thought to contribute to cognitive functions like goal-directed action selection by evaluation of action-outcomes, while the putamen appears to be more limited to sensorimotor coordination (Grahn et al., 2008).

Involvement of the caudate nucleus in cognitive functions is supported by several studies, which found this brain area to be an important node in learning, memory, and feedback-processing (Packard and Knowlton, 2002; Graybiel, 2005). Also, microstimulation of the caudate nucleus has been shown to enhance associative learning in primates (Williams and Eskandar, 2006).

On the other hand, a recent activation likelihood estimation (ALE) meta-analysis across 35 sensorimotor tasks revealed consistent bilateral activation in the putamen (Hardwick et al., 2013). The putamen is thought to synchronize cortical activity that is associated with the selection and propagation of movements (Brown and Marsden, 1998). Specifically, at the initiation of bimanual movements, the putamen showed peak activations bilaterally, which was interpreted as the point in time when specific contributions from higher and lower level cortical motor areas have to be synchronized (Kraft et al., 2007).

By transferring this concept to our results, we observe an age-related shift of the STN coupling away from cognitive-motor (action selection; caudate nucleus) toward basic-motor functions (sensorimotor coordination; putamen). The STN is believed to play a role in motor inhibition and, particularly, in response inhibition and the setting of response thresholds (Aron et al., 2007; Mansfield et al., 2011). Since deterioration of motor control is well known in advanced age (Seidler et al., 2010), a stronger inhibitory influence of the STN on the putamen’s executive motor functions toward more response conservativeness appears reasonable. Previous evidence also points to an increased reliance on cognitive control mechanisms in the context of age-related deficits in lower level motor control (Seidler et al., 2010). Apparently, one aspect of this adjustment process is to release the caudate nucleus from interference by the STN. This might allow a more careful selection of feasible actions by the caudate nucleus.

Age-related changes found in thalamus, insula, and sensorimotor cortex agree well with this concept. In the thalamus, we observed an age-dependent decline of positive FC with STN. The affected prefrontal subregion of the thalamus is involved in cognitive processing like attention, planning, organization, abstract thinking, active memory, and multi-tasking (Eckert et al., 2012). Like in the caudate nucleus, less interference by the STN might enhance cognitive processing in a compensatory manner. Selection of relevant sensory information for integration in the also affected parietal thalamic subregion (Eckert et al., 2012) could similarly be interpreted as another cognitive process that is shut off from STN influences. However, as an alternative hypothesis, a certain amount of influence by the STN on the parietal thalamic subregion might be necessary for sensory filtering. Hence, the age-related decline in FC between both regions could also contribute to changes in sensorimotor performance in the elderly (Seidler et al., 2010).

Our data also revealed an age-related reduction of STN FC with the posterior and central insula. A hierarchical model of the human insula suggests that sensorimotor information initially reaches the insula at its posterior part and is subsequently integrated with emotional and cognitive evaluations in the anterior insula (Craig, 2009; Kurth et al., 2010b). Although these mechanisms are not yet fully understood, it seems very likely that the insula has a superordinate role in interactions between motor, cognitive, and affective functions (Kurth et al., 2010b; Kelly et al., 2012).

Age-related increase in positive STN FC found in the sensorimotor cortex of right face and neck also supports the notion of an age-related shift of the STN coupling away from cognitive-motor toward basic-motor control. To our knowledge, there are no reports of age-related changes in facial motor function. However, hypomimia is a typical symptom in PD patients. Since the prevalence of PD rises with age (de Lau and Breteler, 2006), and incidence of face and neck motor symptoms is known to increase with age (Szewczyk-Krolikowski et al., 2014), these symptoms could be the result of a combination of PD-related oscillatory hyperactivity of the STN (Kuhn et al., 2009) and the observed increased connectivity between STN and corresponding areas in M1.

If the alternative hypothesis is true and motor related activity in sensorimotor cortex triggers STN activity, this network could present a regulatory circuit in which motor activity at the same time stimulates the STN to increase response conservativeness in the motor network. The physiological value of this kind of regulation could lie in preventing initiation of too many new motor programs as long as previous actions are still in the phase of execution. In light of an age-related decline of sensorimotor abilities (Seidler et al., 2010), an increasing need for such a network and, therefore, an enhancement of its connections would seem plausible.

Remarkably, all these age-related changes of positive STN FC were found unilaterally. Bilateral insular clusters were identified only when adopting a more liberal cluster-level threshold (*p* < 0.1). While the lateralization of age-related changes in STN FC with M1 remains especially ambiguous, BOLD-measured activation induced by motor activity was reported to be higher for left, relative to right, basal ganglia in right-handers, regardless of the hand used (Scholz et al., 2000). Although the handedness of participants was not assessed in this study, preponderance of right-handedness is most likely. Potentially, the connection between STN and left putamen is also assigned to motor functions showing left-lateralization at the basal-ganglia level. This could explain why age-related changes in this connection reach detectability only in the left hemisphere.

On the other hand, the right (versus left) striatum appears to be of particular importance for some cognitive processes like active learning (as compared with observational learning), with the caudate nucleus being especially involved in the evaluation of one’s own behavioral outcome (Bellebaum et al., 2012). This underlines the importance of the right caudate nucleus for cognition and our notion of an age-related shift of the STN coupling away from cognitive functions. More specifically, this pattern could mean that the connection between STN and right caudate nucleus is also dedicated to active learning. If the STN drives the caudate nucleus, this might reflect compensating for an age-associated decline in motor learning (Seidler, 2006) by releasing learning processes in the caudate nucleus from interference by the STN. However, if the alternative hypothesis is true and the caudate nucleus drives the STN, learning processes in the caudate nucleus might co-activate the STN for suppression of potentially distracting motor programs. Decline of this connection could therefore be interpreted as a possible cause for the aforementioned age-related worsening of motor learning.

Positive FC between left and right STN increased significantly with age. To our knowledge, implications of such changes in STN interconnectivity for cognition and behavior have not yet been described in the literature. However, it seems possible that the above-mentioned functional segregation of the basal ganglia (right: focus on cognitive functions; left: focus on basic motor functions) does also apply to the subthalamic nuclei. Accordingly, aging would be associated with a decline in this segregation of higher level cognitive functions and lower level motor control, providing a possible neural substrate contributing to the worsening of both motor and cognitive performance with age.

While the cognitive functions that were linked to age-related changes of positive STN FC are all associated with motor functions on a superordinate level, the following age-related changes of negative STN FC are essentially related to non-motor cognitive networks.

### AGE-RELATED CHANGES OF NEGATIVE FC OF THE STN

For the precuneus and the posterior cingulate cortex, we found an age-related decrease in functional anticorrelation (i.e., the neural de-coupling became weaker with age). The posterior cingulate cortex is part of the so-called default-mode network (Leech and Sharp, 2013) and has been implicated in self-reflection (Northoff et al., 2006; Qin and Northoff, 2011; Schilbach et al., 2012). The precuneus has been associated with introspection, memory, and mental imagery (Cavanna and Trimble, 2006). In sum, both posterior medial cortex regions (posterior cingulate cortex and precuneus) subserve cognitive functions that are unrelated to motor functions. Hence, together with the above-mentioned finding that aging influences the functional integration of the STN in motor-related networks, we also observed a reduction of its segregation from non-motor-related networks. The reduced de-coupling of such non-motor regions suggests a reduced inhibition of cognitive processing that is unrelated to STN-related motor control functions and might thus lead to increased interference in advanced age. Interestingly, older adults’ brain activity seems to focus on precuneus and posterior cingulate cortex when thinking about self-relevant agendas (especially duties and obligations), while activity of medial prefrontal cortex in the same context declines with age (Northoff et al., 2006; Mitchell et al., 2009). Hence, it seems conceivable that older adults’ response control (mediated by the STN) has a higher chance of getting disturbed by such contemplations, as compared with younger adults.

### FC CHANGES IN LIGHT OF MICROSTRUCTURAL NEURODEGENERATION

Several factors might underlie the discussed age-related changes in STN FC. Accumulating evidence points toward a central role of blood vessels within neurodegenerative processes. These factors include age-related dysfunction of cerebral autoregulation, structural and functional alterations in cerebral blood vessels including amyloid deposition, neurovascular uncoupling due to astrocyte endfeet retraction, changes in insulin/insulin-like growth factor-1 signaling, and secondary white matter changes (for a review, see Popa-Wagner et al., 2013). Due to high and variable energy demands, neurons are strongly reliant on adequate arterial inflow of oxygen and glucose, while, at the same time, they are highly vulnerable to hypoperfusion because of a very limited energy store (Gouix et al., 2014). An interrelationship between degenerative neuronal degradation and the observed age-related FC changes seems likely. However, current imaging techniques cannot discriminate between FC changes caused primarily by neuronal degradation and those which develop secondarily as adaptive mechanisms.

### LIMITATIONS AND FUTURE DIRECTIONS

Our analysis has the typical limitation of a cross-sectional approach, which necessarily conflates age and cohort effects. Furthermore, although all participants of our analysis were without any record of neurological or psychiatric disorder, the efficiency of this screening at the three sites could have been different. Therefore, sporadic inclusion of participants with sub-clinical cognitive impairments cannot be completely excluded.

Finally, no behavioral data could be obtained from our participants, which would have been useful for testing the presumed cognitive and behavioral effects of the observed age-related FC changes. In future research, it would be desirable to combine the assessment of motor performance with the acquisition of RS-FC data in the same individuals to be able to directly test specific brain–behavior relationships.

## CONCLUSION

Our examination of the global STN FC at “rest” demonstrated a positively coupled network comprising putamen, caudate nucleus, globus pallidus, thalamus, central and posterior insula, anterior and midcingulate cortex, sensorimotor cortex, V1, midbrain, cerebellum and pons. Advanced age was associated with a reduced coupling of the STN with cognitive motor control regions (caudate nucleus, thalamus, and central/posterior insula), increased coupling with lower level motor control regions (sensorimotor cortex and putamen), and reduced anti-correlation with non-motor regions (precuneus and posterior cingulate cortex).

Taken together, these connectivity changes suggest a shift of the STN’s network involvement from more cognitive network coupling (caudate nucleus) toward more motor-related network coupling (putamen, M1). This connectivity changes could potentially provide a compensatory mechanism for the prevention or mitigation of the known degradation of motor performance with age. Apparently, older adults release superordinate motor functions (action selection; motor learning) in the caudate nucleus from interference by the STN and at the same time increase the influence of the STN on the putamen’s and M1’s basic motor functions toward more response conservativeness. Furthermore, the reduced de-coupling of non-motor-related brain areas might indicate a potential cause for age-related decline in motor performance: STN-mediated response control in older adults could thus become more prone to interference from self-relevant but action-irrelevant cognitive processing. In sum, our results suggest that aging affects the functional integration of the STN in motor-related networks and its segregation from non-motor-related networks.

## AUTHOR CONTRIBUTIONS

Simon B. Eickhoff, Robert Langner, Felix Hoffstaedter, and Christian Mathys substantially contributed to the conception and design of the work. All authors substantially contributed to the analysis and interpretation of data for the work. Christian Mathys, Felix Hoffstaedter, and Robert Langner drafted the work. Julian Caspers, Svenja Caspers, Martin Südmeyer, Christian Grefkes, and Simon B. Eickhoff revised the work critically for important intellectual content. All authors finally approved the manuscript version to be published. All authors agree to be accountable for all aspects of the work in ensuring that questions related to the accuracy or integrity of any part of the work are appropriately investigated and resolved.

## Conflict of Interest Statement

The authors declare that the research was conducted in the absence of any commercial or financial relationships that could be construed as a potential conflict of interest.

## Footnotes

1 http://www.who.int/ageing

2 http://www.fil.ion.ucl.ac.uk/spm

3 http://www.fz-juelich.de/ime/spm_anatomy_toolbox, V1.8
